# Silver Nanoparticles Affect Functional Bioenergetic Traits in the Invasive Red Sea Mussel* Brachidontes pharaonis*

**DOI:** 10.1155/2016/1872351

**Published:** 2016-10-05

**Authors:** Ilenia Saggese, Gianluca Sarà, Francesco Dondero

**Affiliations:** ^1^Dipartimento di Scienze ed Innovazione Tecnologica, University of Piemonte Orientale, Via Michel 11, 15121 Alessandria, Italy; ^2^Dipartimento di Scienze della Terra e del Mare, University of Palermo, Viale delle Scienze Ed. 16, 90128 Palermo, Italy

## Abstract

We investigated the functional trait responses to 5 nm metallic silver nanoparticle (AgNPs) exposure in the Lessepsian-entry bivalve* B. pharaonis*. Respiration rate (oxygen consumption), heartbeat rate, and absorption efficiency were evaluated across an 8-day exposure period in mesocosmal conditions. Basal reference values from not-exposed specimens were statistically compared with those obtained from animals treated with three sublethal nanoparticle concentrations (2 *μ*g L^−1^, 20 *μ*g L^−1^, and 40 *μ*g L^−1^). Our data showed statistically significant effects on the average respiration rate of* B. pharaonis*. Moreover, complex nonlinear dynamics were observed as a function of the concentration level and time. Heartbeat rates largely increased with no acclimation in animals exposed to the two highest levels with similar temporal dynamics. Eventually, a decreasing trend for absorption efficiency might indicate energetic constraints. In general, these data support the possible impact of engineered nanomaterials in marine environments and support the relevance of functional trait assessment in present and future ecotoxicological studies.

## 1. Introduction

The quick development of nanotechnology is posing concerns on the environmental impact of engineered nanomaterials (ENMs) into delicate environments such as marine coastal areas. The production and commercial use of ENMs have been linearly increased during the last decade and involve the following: personal care, cosmetics, fabrics and textile, household appliances, electronics and computers, health and medicine, renewable energies, and environmental remediation [1]. According to the Nanotechnology Consumer Products Inventory [2], currently, there are 1841 ENM-containing products in the consumer marketplace, of which 438 (24%) contain nanosilver. Silver is a xenobiotic metal widely used in industrial applications (more than 60% of the metal demand) such as chemical catalysis, batteries, photography, electronics, and brazing and soldering [3]. Due to its antimicrobial properties it has been largely employed as additive in personal care products, coatings, paints, fabrics, food, and beverage. Nowadays, most of the latter applications as well as those targeted to health, fitness, and household use are based on silver ENMs [2]. Indeed, silver at the nanoscale level shows distinctive physicochemical properties and biological activities like excellent conductivity, chemical stability, and increased catalytic activity [4]. These distinctive properties differ significantly from those of larger particles and have made silver nanoparticles (AgNPs) extremely attractive for the production of consumer materials and, therefore, the investigation of their environmental fate and toxicological properties [4, 5]. Nanosilver predicted environmental concentrations (PECs) are yet affected by uncertainty since different approaches in modeling and measurement methods complicate validation. Gottschalk and coworkers [6] reviewed the outcome from different predictive models. For superficial waters they indicated a wide silver range spanning from 10^−4^ to 10^0^ ppm with a median value around 10^−2 ^ppm. Waste water treatment plants are able to capture most (nano)silver into an insoluble sulfide form [7]; however, ENMs may enter into the environment through different routes such as outdoor urban sources [8] that are not necessarily intercepted by treatment plants. Air transport and in general the hydrogeological cycle have a primary role in ENM run-off and transport, thus, making marine ecosystems the terminal sinks [9]. PECs for seawater environments are practically unknown.

The antimicrobial as well as toxic effects of silver are attributed to the Ag^+^ ion that is a strong electrophile reacting with most macromolecules containing S, O, and N [5, 10, 11]. The toxicity of AgNPs in the environment is certainly a function of the silver metal speciation that highly depends on the environment itself but also intrinsic characteristics such as particle sintering, coating, surface potential, size, and Ag^+^ dissolution rate [12–14]. It is widely recognized that Ag-ENMs can exert toxic effects on aquatic organisms such as algae, mollusks, crustaceans, and fish for which classical ecotoxicological endpoints have been evaluated [1, 15]; however, very little is known about marine species [16].

Marine bivalves are model species to study ENM effects. Since they are filter-feeder organisms, they bioaccumulate toxicants either in dissolved or in particle-adsorbed forms in the water column [17, 18]. However, the main routes of NPs uptake and the level at which they can penetrate into the organism are not completely known [5]. In the bivalve* Mytilus edulis* Moore [17] showed that the principal process for the translocation of polystyrene NPs across the membrane is endocytosis. Regarding AgNP data on toxicological effects on bivalves, this information is sparse. According to Zuykov et al. [19] AgNPs can be accumulated in the extrapallial fluid of the blue mussel* Mytilus edulis* and concentrate mostly in ganglia. Gomes et al. [20] reported an oxidative stress syndrome in soft tissues of* Mytilus *spp. with induction of heavy metal binding proteins in gills. In the oyster* C. virginica* there were embryo toxicity and effects on lysosomal integrity of hepatopancreas cells [21]. In this context, functional traits such as food assimilation, respiration, and heartbeat rates [22] may represent a straightforward and useful approach to investigate AgNPs metabolic and bioenergetic effects. Here we report the effects of AgNPs on main functional traits of a new proposed model species, the Lessepsian-entry bivalve* Brachidontes pharaonis* [23, 24]. Indeed, we tested the sensitivity of* B. pharaonis* to submicromolar amounts (0–40 *μ*g L^−1^) of a 5 nm commercial AgNP in an 8-day exposure experiment carried out in laboratory under mesocosmal conditions. The response variables observed were respiration rate (RR), heartbeat rate (HBR), and absorption efficiency (AE).

## 2. Material and Methods

### 2.1. Animals and Treatments with Silver Nanoparticles

Specimens of* Brachidontes pharaonis* were collected from the Ettore Pond of Stagnone di Marsala (Trapani, Western Sicily, 37° 52′ north; 12° 28′ east). Once collected, mussels were brought back to the laboratory in controlled conditions of temperature (16°C) and humidity (100%). They were cleaned from epibionts and moved into tanks at 0.5 L per animal in filtered recirculating seawater at 20°C. Organisms were acclimated in laboratory conditions for 15 days at 20°C, 36‰ salinity, and pH 8.2 ± 0.1. The total number of animals used in this experiment was 360, with a size ranging from 22 to 27 mm. Both during the acclimation and during experimental periods, organisms were fed three times per day (ad libitum) with fresh cultures of* Isochrysis galbana* at an initial titer of 15,000 cells mL^−1^. Animals were divided into 4 experimental groups of 90 specimens, with 3 independent tanks per group. Each group corresponded to a different exposure level to silver nanoparticles: 0 (not-exposed, reference control), 2, 20, and 40 *μ*g L^−1^. This concentration range is compatible with previous studies on AgNPs [20, 25].

Silver nanoparticles (5 nm average diameter) with an alkane coating were supplied by AMEPOX (Lodz, Poland) in a stable ultrapure-water solution at 1 g L^−1^. This material has been already used in a large ENM toxicity-testing framework within the NanoFATE project (https://wiki.ceh.ac.uk/display/nanofate/Home) [26–29].

### 2.2. Respiration Rate

Metabolic respiration rates were evaluated as oxygen consumption according to a reliable procedure already tested in companion papers [30, 31]. Measurements were carried out every 2 days on six randomly selected specimens per treatment (0, 2, 20, and 40 *μ*g L^−1^). Briefly, single* B. pharaonis* specimens were placed in glass respirometric chambers (0.5 L) containing filtered air-saturated seawater. Magnetic stirring ensured water mixing within the chamber, while oxygen reduction was measured by means of computer-assisted optical oxygen-meter probes (FireSting O_2_, PyroScience GmbH, Aachen, Germany) in four different chambers (one per condition) simultaneously. The rate of oxygen consumption was then calculated according to [32]: RR (O_2_ *μ*moles/h) = [*C*(*t*_0_) − *C*(*t*_1_)]·(*Vr*) · 60/(*t*_1_ − *t*_0_), where *t*_0_ and *t*_1_ represent start and finish times (min) of the measurement period; *C*(*t*) is concentration of oxygen in the water (*μ*moles O_2_ L^−1^) at time *t*; and *Vr* is volume of respirometer minus the animal. Respiration rate correction for body mass was carried out using dry weight [32].

### 2.3. Heartbeat Rate

Heartbeat rate (HBR, beats/min) was evaluated by means of a noninvasive cardioplethysmographic technique [22, 33]. To record the mussel heartbeat, infrared sensors were glued on the left side of the mussel shell, just below the umbone. To avoid stress to the organism, sensors were positioned the day before the measurement. The heartbeat signals obtained were amplified, filtered, and then detected by means of a portable oscilloscope PicoScope 236 (Pico Technology Ltd., UK) connected to a laptop computer equipped with PicoScope 6.0 software. During the experimental session, HBR was recorded at intervals of 10 minutes per mussel. HBR values for each animal were obtained as the average of 3 randomly selected views of the 10-min measurement. Measurements were made every 2 days on six randomly selected individuals per treatment.

### 2.4. Absorption Efficiency

Food absorption efficiency was measured by comparing the proportions of organic matter in the algal cells and mussels feces according to the equation of Conover [34]: AE = (*F* − *E*)/[(1 − *E*)*F*], where *F* is a relationship between dry weight and ash-free dry weight of algal food while *E* is a relationship between dry weight and ash-free dry weight of fecal pellets. Fecal pellets from each treatment were collected daily and placed in separate vials. Collected feces were filtered on preweighted glass fiber filters (Whatman GF/C, 0.45 *μ*m) and washed with 0.5 M ammonium formate to remove salts [35]. Then, samples were dried at 100°C for 48 h and dry weights were recorded immediately after cooling in a desiccator. Eventually, samples were ashed in a furnace at 450°C for 2 h and then reweighted to obtain the ash-free dry weight. The results are the average of six different measurements per condition.

### 2.5. Statistical Analysis

An analysis of variance (ANOVA) was performed using R (software version 2.15.1) to test the dependence of RR, HBR, and AE on AgNP concentration (CONC, fixed, 4 levels) and time (TIME, fixed, 5 levels). The assumption of homoscedasticity was tested using Cochran's *C* test. Post hoc comparisons were made using the Student-Newman-Keuls test (SNK-test). The alpha values are reported on each table or figure.

## 3. Results and Discussion

**Figure 1:**
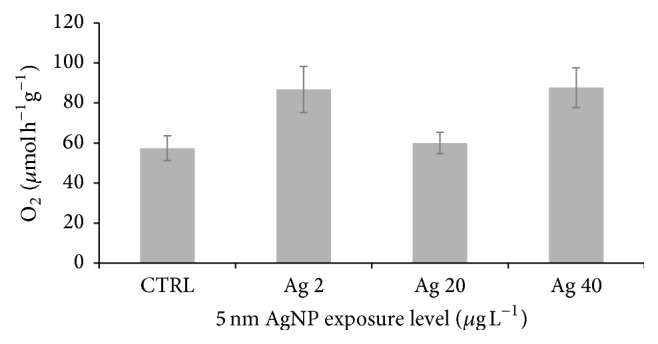
Effects of 5 nm AgNP on average* B. pharaonis* respiration rates (RR). Shown is the normalized average (±SEM) respiration rate (*μ*mol h^−1^ g^−1^) across the 8-day exposure period. The ANOVA post hoc test did not show statistically significant differences between the control not-exposed samples and the AgNP treated ones (TNK-test *P* > 0.05).

**Figure 2:**
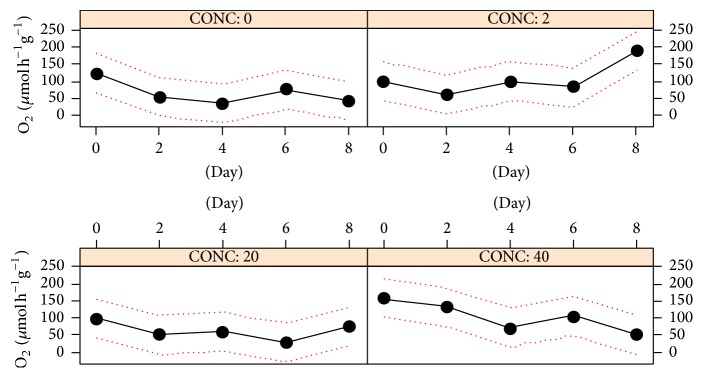
*B. pharaonis* respiration rates (RR) dynamics. The plot shows respiration rates (RR, *μ*mol h^−1^ g^−1^) versus AgNP concentrations (*μ*g L^−1^). Continuous line: average RR; dotted-line, 1-SD confidence interval.

The exposure to 5 nm AgNPs significantly influenced the overall* B. pharaonis* respiration rate (RR) (Table 1, Figure 1). This is the result of complex dynamics especially occurring in specimens treated with 2 and 40 *μ*g L^−1^ AgNP during the 8-day exposure (Figure 2). These samples showed opposite temporal trends and seem to reflect different compensation strategies to discrete nanoparticle amounts. The lowest concentration, in fact, determined a progressive increase of oxygen consumption in time and by contrast the highest Ag level caused an increase of respiration during the first part of the exposure, then, followed by a constant decrease to an average value of about 49 *μ*mol h^−1 ^g^−1^ similar to that of control (Figure 2). The intermediate Ag level displayed negligible effects with respect to control. The average individual respiration rate of control specimens observed in this study was around 10 *μ*M mol O_2_ h^−1^. This measure was compatible with previous values reported in the same species [24].

**Figure 3:**
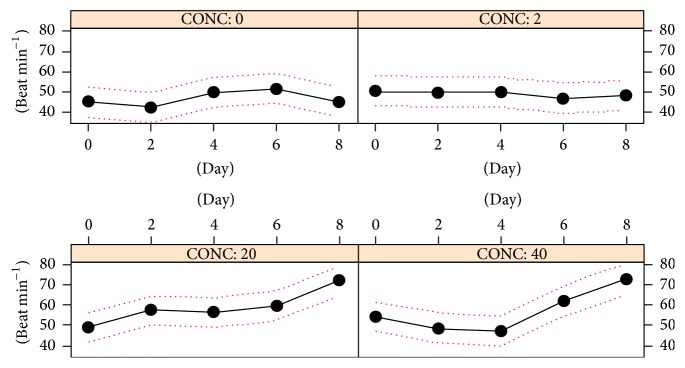
*B. pharaonis* heartbeat rates (HBRs) dynamics. The plot shows heartbeat rates (HBRs, beat min^−1^) versus AgNP concentrations (*μ*g L^−1^). Continuous line: average HBR; dotted-line, 1-SD confidence interval.

5 nm AgNPs had statistically significant effects on the heartbeat rate (HBR) (Table 2).  Figure 3 shows the temporal trends obtained across the 8-day exposure period. These are consistent with an increase of HBR in samples exposed to the highest silver levels, that is, 20 and 40 *μ*g L^−1^. The ANOVA output (Table 3) clearly indicates that HBRs are higher in the latter samples than in control animals as well as those exposed to 2 *μ*g L^−1^ AgNPs (Table 3).

Basal HBRs were similar to values previously reported [33].

**Figure 4:**
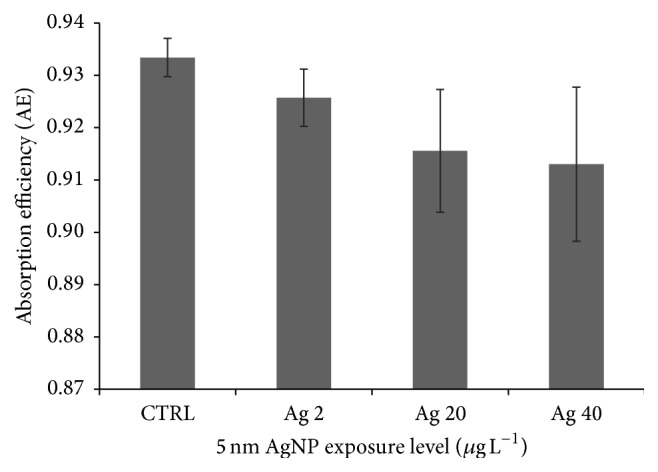
*B. pharaonis* absorption efficiency (AE). AE average (±SEM) values for the effects of 5 nm AgNP exposure are depicted. The decreasing trend observed was not statistically significant (1-way ANOVA, *P* = 0.18).

Finally, no statistically significant effects were found in absorption efficiency (AE) of mussels exposed to AgNPs (*P* > 0.05). However, Figure 4 depicts a decreasing AE trend across the silver concentration gradient.

Respiration and heartbeat rates are two important physiological traits in ectothermic organisms, such as bivalves. They may reflect the energetic budget availability thus informing on higher organizational levels, that is, growth and fecundity [36–38]. For this reason RR and HBR have been used to predict physical and chemical stress effects for a long time. Numerous researchers have reported a dose dependent decrease of HBR for the chronic effects of heavy metal exposure, such as copper, however, starting from concentrations higher than 50 *μ*g L^−1^ [39–41]. It should be pointed out that, in the present study, silver was continuously presented to mussel in a nonreactive form, that is, the elemental state. Preliminary data obtained by our research group suggest a sudden precipitation of AgNP in seawater and a relatively low dissolution rate of silver to Ag^+^ ions (unpublished observations). This means that the actual bioavailable as well as active silver fraction in the water column was certainly below the nominal concentration used, shifting the actual tested range within a genuine ppb scale. The results presented in this work indicate that AgNPs can affect two important physiological processes of the marine bivalve* B. pharaonis*, that is, metabolic respiration rate and heartbeat rate. HBRs showed a consistent increase in samples exposed to the two highest AgNP levels (20 and 40 *μ*g L^−1^) (Figure 3). This finding is only in apparent contrast with the aforementioned previous studies, since our data can be reconciled by hormesis. Hormesis represents a relatively new paradigm in toxicology for which responses to different kinds of toxic agents (or drugs) behave nonlinearly within a threshold level, usually very low and thus disregarded in common toxicological frameworks [42, 43]. Indeed, in specimens of* B. pharaonis *and* Mytilaster minimus* subjected to relatively high and recurrent levels of disturbance—such as hypersalinity or hyperthermia—a sharp and continuous increase of HBR has been described before occurring of severe bradycardia [33, 44]. We, therefore, can conclude that the observed HBR changes are likely to represent a compensation strategy to counteract the disturbance level escalation, for example, increasing silver burdens accumulating into mussel soft tissues across the exposure.

The outcome of this study showed a more complex response pattern to 5 nm AgNPs concerning the respiration rate (RR) (Figures 1 and 2). ANOVA pointed out significant effects for the variable concentration (CONC, Table 1) but nonlinear responses were observed (Figure 1). In 40 *μ*g L^−1^ exposed animals RR dynamics displayed a fair increase followed by acclimation to control values. This is consistent with the acclimation properties of RR, as reported for moderate temperature shift by Widdows [45]. Instead, no response was observed at the intermediate dose and a delayed RR intensification was seen at 2 *μ*g L^−1^ AgNPs. Similar results were seen in the bivalve* Mytilus galloprovincialis* Lam. exposed to ZnO NPs for 12 weeks, that is, low-to-intermediate doses did not render a perfect dose response pattern on some physiological and behavioral traits such as RR and survival [46]. These findings confirm that, in general, ENM toxicity should be a function of factors other than the mere nominal tested concentration since the evolution and fate of NPs in the tested environment can eventually influence the internal dose. To this end, it is worth noting that Mullera et al. [47], as part of the same toxicological framework studied by Hanna et al. [46], reported a good correlation between the internal ZnO level and RR. Furthermore, as previously discussed for HBR, nonlinearity (sensu hormesis) represents an intrinsic item of the toxicological response; therefore, RR fluctuations across increasing AgNP doses should be considered a genuine feature of the physiological adaptation process.

Energy flows through the food chain in ecosystems and ecosystems themselves rely on that energy [48]. Our data on AgNP exposure indicated a decreasing trend for food absorption efficiency, although this effect was not statistically significant (Figure 4). This result is not trivial since, as recently demonstrated by our research group [24], even a small reduction in the efficiency to assimilate food may give rise to important impacts in terms of life history trait's magnitude (fecundity and growth rate) or energy allocated to byssus quality and quantity [36], eventually increasing the likelihood of subpopulation dislodgment and/or extinction.

While we are aware that mesocosmal conditions can be highly controlled to generate reliable outcomes [24, 49–51], present results, despite their relevance in an ecotoxicological framework of ENM toxicity assessment, showed that a relatively short exposure period (8 days) is probably not enough to delineate the entire dynamics that characterize* Brachidontes* sp. response to stress. Therefore, longer exposure period should be envisaged in further studies.

## 4. Conclusions

This work demonstrates that in marine bivalves basic physiological functions are impacted by relatively low concentrations of AgNP, an emerging contaminant, and supports the use of functional bioenergetics into mechanistic frameworks able to inform on ecological niche changes starting from simple individual responses [52].

## Figures and Tables

**Table 1:** ANOVA details for the effects of 5 AgNP exposure on *B. pharaonis* normalized respiration rate (RR).

Source	DF	MS	*F*	*P*
CONC	3	8570.4	3.62	*∗*
TIME	4	5490.6	2.32	ns
CONC × TIME	12	4336.8	1.83	ns
Residuals	40	2365.7		

Factors tested for dependence were concentration (CONC) and exposure time (TIME). Shown are DF, degree of freedom; MS, mean square; *F*, *F*-test result; and *P*, *P* value [*∗* = *P* ≤ 0.05; and ns = no significant difference (*P* > 0.05)].

**Table 2:** ANOVA details for the effects of AgNP exposure on *B. pharaonis* heart beat rate (HBR).

Source	DF	MS	*F*	*P*
CONC	4	215.2	5.342	*∗∗*
TIME	3	510.8	12.679	*∗∗∗*
CONC × TIME	12	124.8	3.098	*∗∗*
Residuals	40	40.3		

Factors tested for dependence were concentration (CONC) and exposure time (TIME). Shown are DF, degree of freedom; MS, mean square; *F*, *F*-test result; and *P*, *P* value [*∗∗* = *P* ≤ 0.01; *∗∗∗* = *P* ≤ 0.001; and ns = no significant difference (*P* > 0.05)].

**Table 3:** ANOVA post hoc comparison for HBR.

	CONC	0	0	0	0	0	2	2	2	2	2	20	20	20	20	20	40	40	40	40	40
CONC ×	TIME	0	2	4	6	8	0	2	4	6	8	0	2	4	6	8	0	2	4	6	8

0	0	—																			
0	2	ns	—																		
0	4	ns	ns	—																	
0	6	ns	ns	ns	—																
0	8	ns	ns	ns	ns	—															
2	0	ns	ns	ns	ns	ns	—														
2	2	ns	ns	ns	ns	ns	ns	—													
2	4	ns	ns	ns	ns	ns	ns	ns	—												
2	6	ns	ns	ns	ns	ns	ns	ns	ns	—											
2	8	ns	ns	ns	ns	ns	ns	ns	ns	ns	—										
20	0	ns	ns	ns	ns	ns	ns	ns	ns	ns	ns	—									
20	2	ns	ns	ns	ns	ns	ns	ns	ns	ns	ns	ns	—								
20	4	ns	ns	ns	ns	ns	ns	ns	ns	ns	ns	ns	ns	—							
20	6	ns	ns	ns	ns	ns	ns	ns	ns	ns	ns	ns	ns	ns	—						
20	8	*∗∗*	*∗∗∗*	*∗*	*∗*	*∗∗*	*∗*	*∗*	*∗*	*∗∗*	*∗∗*	*∗∗*	ns	ns	ns	—					
40	0	ns	ns	ns	ns	ns	ns	ns	ns	ns	ns	ns	ns	ns	ns	ns	—				
40	2	ns	ns	ns	ns	ns	ns	ns	ns	ns	ns	ns	ns	ns	ns	*∗∗*	ns	—			
40	4	ns	ns	ns	ns	ns	ns	ns	ns	ns	ns	ns	ns	ns	ns	*∗∗*	ns	ns	—		
40	6	ns	ns	ns	ns	ns	ns	ns	ns	ns	ns	ns	ns	ns	ns	ns	ns	ns	ns	—	
40	8	*∗∗∗*	*∗∗∗*	*∗∗*	*∗*	*∗∗∗*	*∗*	*∗∗*	*∗∗*	*∗∗*	*∗∗*	*∗∗*	ns	ns	ns	ns	ns	*∗∗*	*∗∗*	ns	—

SNK-test and *P* value [*∗* = *P* ≤ 0.05; *∗∗* = *P* ≤ 0.01; *∗∗∗* = *P* ≤ 0.001; and ns = no significant difference (*P* > 0.05)].
